# CT based radiomic approach on first line pembrolizumab in lung cancer

**DOI:** 10.1038/s41598-021-86113-5

**Published:** 2021-03-23

**Authors:** Marta Zerunian, Damiano Caruso, Alberto Zucchelli, Michela Polici, Carlo Capalbo, Marco Filetti, Federica Mazzuca, Paolo Marchetti, Andrea Laghi

**Affiliations:** 1 Radiology Unit, Department of Medical-Surgical Sciences and Translational Medicine—“Sapienza”, University of Rome, Sant’Andrea University Hospital, via di Grottarossa 1035, 00189 Rome, Italy; 2 Oncology Unit, Department of Clinical and Molecular Oncology—“Sapienza”, University of Rome, Sant’Andrea University Hospital, via di Grottarossa 1035, 00189 Rome, Italy; 3 IDI-IRCCS, Via Monti di Creta 104, 00167 Rome, Italy

**Keywords:** Prognostic markers, Data processing, Image processing, Software, Statistical methods, Tumour biomarkers, Non-small-cell lung cancer

## Abstract

Clinical evaluation poorly predicts outcomes in lung cancer treated with immunotherapy. The aim of the study is to assess whether CT-derived texture parameters can predict overall survival (OS) and progression-free survival (PFS) in patients with advanced non-small-cell lung cancer (NSCLC) treated with first line Pembrolizumab. Twenty-one patients with NSLC were prospectively enrolled; they underwent contrast enhanced CT (CECT) at baseline and during Pembrolizumab treatment. Response to therapy was assessed both with clinical and iRECIST criteria. Two radiologists drew a volume of interest of the tumor at baseline CECT, extracting several texture parameters. ROC curves, a univariate Kaplan-Meyer analysis and Cox proportional analysis were performed to evaluate the prognostic value of texture analysis. Twelve (57%) patients showed partial response to therapy while nine (43%) had confirmed progressive disease. Among texture parameters, mean value of positive pixels (*MPP*) at fine and medium filters showed an AUC of 72% and 74% respectively (*P* < 0.001). Kaplan-Meyer analysis showed that *MPP* < 56.2 were significantly associated with lower OS and PFS (P < 0.0035). Cox proportional analysis showed a significant correlation between *MPP4* and OS (*P* = 0.0038; HR = 0.89[CI 95%:0.83,0.96]). In conclusion, *MPP* could be used as predictive imaging biomarkers of OS and PFS in patients with NSLC with first line immune treatment.

## Introduction

Lung cancer is the leading cause of cancer death in both men and women in the United States^1^ and in Europe^2^. Non-small cell lung cancers (NSCLCs) account for 85% of lung cancers^3^, and more than 50% of patients with primary NSCLC present stage IV disease at diagnosis^4^. The standard of care of stage IV NSCLC involves platinum-based doublet chemotherapy and targeted therapies (some of which not yet available outside of clinical trials) in the presence of sensitizing alterations in EGFR, ALK, ROS-1, and BRAF^5^ or, more recently, NTRK gene fusions, RET rearrangements^6^, MET amplification or mutation, ERBB2 (HER2) mutation.

Alongside this approach, a new therapy targeting the immune system has been developed in recent years: these drugs are monoclonal antibodies that inhibit the programmed cell death protein-1/programmed cell death-ligand 1 (PD-1/PD-L1) immune check-point, preventing the inhibition of the tumor cell-killing function of T-lymphocytes^7^. Currently, several immune checkpoint inhibitors (with or without chemotherapy), such as Pembrolizumab, Nivolumab (with or without ipilimumab), Atezolizumab, and Durvalumab have been approved or are in the process of being approved by the FDA and EMA in first or subsequent-line NSCLC treatment or in different clinical scenarios. Pembrolizumab for non-small cell lung cancer patients with PD-L1 expression ≥ 50% was the first approved immune checkpoint inhibitors first-line therapy^8^.

The response to immunotherapy is extremely variable, with some patients not responding or having a disease progression during immunotherapy^9^, suggesting that there are some individual characteristics still unknown that may determine the effectiveness of such treatment. The identification of these characteristics would improve the selection of patients who would benefit from immunotherapy or in which it is necessary to identify early different ways of using immunotherapy.

Texture analysis (TA) is a recent biomarker that allows to assess quantitative parameters, extrapolated from Imaging (e.g. Computed Tomography, Magnetic Resonance, Positron Emission Tomography) and related to the distribution of pixels and voxels of gray, that correlate with some biological characteristics of the tumor such as heterogeneity^10–13^.

Previous works have shown how some TA parameters correlate with survival in patients treated with the novel immunotherapeutic approach or TKIs for different malignancies such as melanoma and colorectal liver metastasis^14,15^ and results are encouraging.

Up to know, for the best of our knowledge, no studies have performed this evaluation in patients with NSCLC treated with first line Pembrolizumab. In order to avoid possible interferences on the morphological evaluation in patients treated at different times of the disease, we decided to restrict our analysis to NSCLC patients treated on the front line with pembrolizumab, in accordance with the registered indications. Thus, the aim of our study is to evaluate the prediction of quantitative TA parameters before treatment and their correlation with survival endpoints, in patients with stage IV NSCLC treated with first line Pembrolizumab.

## Methods

### Study population

The protocol for fist line Pembrolizumab used in advanced lung cancer, has been approved by the Ethics Committee of Italian Medicine Agency (AIFA, n. 1303/19/05/2017) and all patients were prospectively included in accordance with Helsinki declaration. After obtaining informed consent, 53 patients who had newly diagnosed IV stage lung cancer were prospectively enrolled from July 2017 to June 2019 and received Pembrolizumab as first line immunotherapy.

Inclusion criteria were as follows: (a) inoperable IV stage NSCLC according to TNM 8th edition^16^; (b) histologically proven NSCLC with PD-L1 mutation expression > 50% for eligibility to Pembrolizumab treatment as first line; (c) no prior oncologic treatment; (d) standardized total-body contrast enhanced CT (CECT) performed at baseline and follow-up; (e) CECT solid lung lesion assessable for texture analysis. Were excluded patients if they matched the following characteristics: (a) non-available baseline CECT; (b) incomplete CT protocol (i.e. lack of thoracic venous phase); (c) prior chemotherapy administration (i.e. to avoid possible changes in the tumoural tissue due to previous treatment that might alter data extraction); (d) presence of excavated lung lesion non-suitable for texture segmentation due to the presence of central large area filled with gas, with no texture data extractable, and thin solid peripheral wall non-sufficiently representative for data extraction.

**Figure 1:**
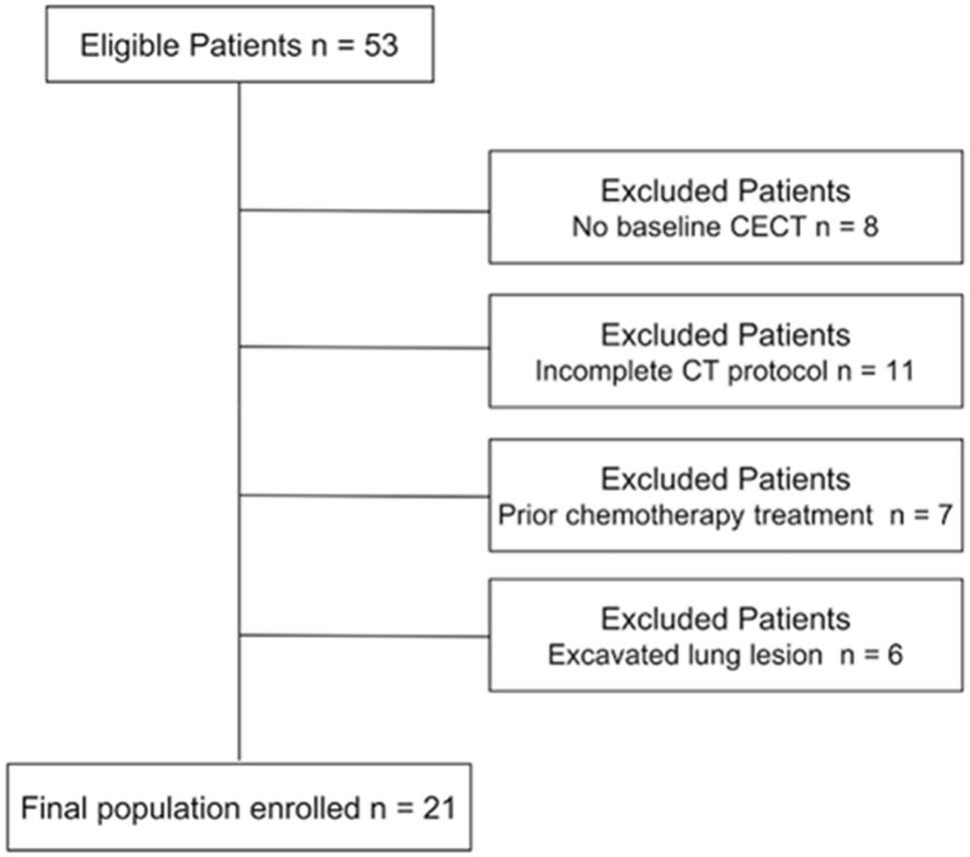
Final population enrollment flow-chart.

From the initial population of 53 patients enrolled were excluded: eight patients without available pretreatment CECT evaluation, 11 patients without thoracic venous acquisition, seven patients who underwent prior chemotherapy treatment and six patients with lung lesion non- clearly delineable for the radiomic analysis as described in the flowchart (Fig. 1).

Other patient data were included as follows: age, gender, smoking habit, Eastern Cooperative Oncology Group (ECOG) performance status score, date of Pembrolizumab treatment beginning, date of CT examinations, lesion chosen for texture analysis, iRECIST conclusion, and eventual time of progression and or death were also included.

All Patients enrolled started a first-line immunotherapy with Pembrolizumab within three to six weeks from the baseline CECT, a humanized antibody targeting the PD-1 receptor of lymphocytes, at standard dose of 200 mg i.v. every 3 weeks according to AIOM-ESMO guidelines^17^.

### CT examination

All CT examinations were performed using a 4 cm detector width CT (Revolution EVO, GE Medical Systems, Milwaukee, WI, USA). All patients were studied in supine position, in craniocaudally direction, with a z-axis coverage from the diaphragmatic dome to the upper iliac bone margin for unenhanced and arterial phase, from the jugular to the pubic symphysis for the venous phase and from C2 vertebra to the cranial verge for the brain. For the specific purpose of this study only the venous phase was analyzed.

Acquisition parameters of the venous phase were as follows: tube voltage, 120 kVp; mAs range 130–260 with tube current modulation SMART mA, GE Healthcare; section collimation, 64 × 0.625 mm; helical pitch, 0.984; scan time per spiral, 0.6 s; image reconstruction thickness, 1.25 mm with standard soft tissue reconstruction and bone filter reconstruction. Images were reconstructed by using the routine-dose and reduced-dose scan series with Filtered Back Projection at 80% and Iterative Reconstruction at 20% (ASiR-V, GE Healthcare, Milwaukee, USA).

The Lean Body Weight related James formula^18,19^ was applied to calculate personalized amount of contrast medium per patient using iso-osmolar non-ionic contrast medium (Iodixanol 320 mg I/mL, Visipaque 320; GE Healthcare, Cork, Ireland)^20^. The contrast medium was intravenously administered through an 18 or 16-gauge antecubital intravenous access at a flux of 3.5 mL/s followed by a 50 mL saline flush at the same flow rate. Scan timing was determined by an automatic bolus tracking (SmartPrep, GE Healthcare) technique by placing a region- of-interest (ROI) at 120 kV in the abdominal aorta at the level of the celiac tripod. The threshold for scan initiation was set at an attenuation of 150 Hounsfield Units. After contrast medium injection a multiphasic acquisition protocol was applied as follows: the late arterial phase was acquired 15 s after reaching the threshold, while the portal venous phase were acquired 70 s after reaching the threshold. After 180 s from the threshold, the brain acquisition was performed.

### Tumor volumetric segmentation and Radiomic feature extraction

Heterogeneity of lung tumors was assessed with TexRAD, a proprietary software algorithm (TexRAD Ltd)^21^. The segmentation was performed in consensus by a two operator (M.P. and A.Z. with 5 and 8 years of experience in texture analysis and Thoracic Oncology respectively) that drawn a volumetric region of interest (VOI) of the main lung lesions appreciable on axial portal-venous pre-treatment CECT. Radiomic features were then extrapolated from the obtained VOIs using the image histogram (first order) statistical method that refers to the frequency of the intensity of pixels. The in-plane filtration step used a Laplacian of Gaussian spatial band-pass filter to produce a series of derived images highlighting features at different anatomic spatial scales ranging from fine to coarse texture. The scale was selected by altering the spatial scale filter (SSF) value between 0 and 6 to extract CT intensity features of different sizes varying between fine (0–2 mm), medium (3–4 mm) and coarse (5–6 mm) filters. Heterogeneity within this VOIs was quantified with and without image filtration using the following histogram parameters: *Mean*, *Standard Deviation*, *Entropy*, *Kurtosis, Skewness*, and *Mean value of positive pixels* (*MPP*). As described by Miles KA and colleagues in 2013^22^, *Mean* refers to the average value of the pixels included in the region or volume of interest, *Standard Deviation* represents a measure of variation from the *Mean* values. *Entropy* quantifies the irregularity of gray-level distribution. *Kurtosis* expresses peakedness and tailedness of the histogram, it is inversely related to the number of features highlighted and increases by intensity variations in highlighted features. *Skewness* evaluated pixel distribution asymmetry and *MPP* express the average brightness of positive pixel values within the image.

### Follow-up and clinical endpoints

All patients underwent clinical, biological, and radiological follow-up according to institution protocol, every 10–12 weeks.

Radiological follow-up consisted of total body CECT scans with a standard protocol as abovementioned and for the purpose of the study the analysis was performed on the baseline CECT. Radiological reports were based on iRECIST criteria^23^.

To perform the analysis two main temporal and clinical endpoints were chosen: overall survival (OS) and progression-free survival (PFS). OS was defined as the time from initiation of immunotherapy with Pembrolizumab to death while PFS was defined as the time from the first administration of Pembrolizumab to clinical and radiological progression in accordance to the prior described criteria. In case of discordance between clinical and radiological progression, the clinical parameters including ECOG score, clinical symptoms (improvement of disease-related symptoms) or Pembrolizumab toxicity were considered^23,24^.

For patients alive at the end of follow-up OS were considered censored as well as for patients recurrence free and PFS.

### Statistical analysis

Statistical analysis was performed using SPSS version 21.0 (SPSS Inc. Chicago, IL) and MedCalc Statistical Software version 17.9.7 (MedCalc Software bvba, Ostend, Belgium), and *P* values < 0.05 were considered statistically significant.

All texture features were analyzed with Receiver operating characteristic (ROC) curves and the Area under the curve (AUC) was calculated for predicting the performance of the texture analysis. Then, the significant radiomic features were further analyzed to assess the texture parameter as predictor of outcome, with univariate Kaplan–Meier to identify an optimal threshold separating patients with good and poor prognosis, using non-parametric log-rank test.

To investigate possible associations between texture parameter and OS a Simple Cox proportional hazard regression analysis was performed. The analysis is considered exploratory due to no adjustments for multiple testing carried out.

### Ethical approval and consent to participate

This prospective study was IRB approved and informed consent was obtained from all patients following Helsinki declaration.

### Consent for publication

Written consent for publication of images was obtained from Patients.

## Results

### Study population and follow-up

The final population included 21 patients, 14 males and 7 females (median age 59 years, range 45–82 years), with a mean BMI of 23.74 ± 4.03. Of the final population enrolled 16 patients (11 males and 5 females) were current smoker, four (three males and one females) were former smokers, while one female had no smoking habit. Twenty-one patients had NSCLC including 18 adenocarcinoma and three squamous cell carcinomas. All patients resulted wild type for ALK, and all except one were wild type for EGFR; one patient showed exon 19 deletion. Recorded ECOG score was 0 for nine patients, one for eleven patients and two for one patient. All clinical and demographic parameters are displaced in Table 1.

**Table 1:** Demographic and clinical patients’ characteristics, tumor characteristics.

Characteristics	Number of patients (n = 21)	(%)
Age	Mean 59 years (45–82)	–
Gender (male)	14	66
BMI	23.74 ± 4.03	–
**Smoke habit**		
Current smoker	16	76
Former smoker	4	19
Non-smoker	1	5
**ECOG score**		
0	9	43
1	11	52
2	1	5
**NSCLC type**		
Adenocarcinoma	18	86
Squamous cell ca	3	14
**ALK**		
Wild type	21	100
**EGFR**		
Wild type	20	95
Exon 19 deletion	1	5
PD-L1 expression (> 50%)	21	100
Brain metastasis	6	29
Bone metastasis	5	23
Liver metastasis	1	5

According to clinical parameters and iRECIST, 12 (57%) patients showed non-progressive disease to therapy (including immune- partial response, complete response and stable disease) while 9 (43%) patients showed progression of the disease.

Median OS and PFS were 270 days (range 90–720) and 120 days (range 30–240), respectively. Nine out of 21 patients (42%) died during the follow-up.

### Tumor volumetric segmentation and Radiomic feature extraction

The pretreatment ROC curve analysis of the 36 texture features (6 texture parameters per 0–6 SSF), is displaced in Table 2.

**Table 2:** Table shows performance of pretreatment contrast enhanced CT Texture features at different Spatial Scale Filters (SSF) expressed by Receiving Operating Curve (ROC) analysis with Area Under the Curve (AUC), sensitivity, specificity, cut-off criteria and P values (significant P values < 0.05 are highlighted with asterisks).

Texture Parameter	SFF	AUC	Sensitivity	Specificity	Cut-off	*P* value
Mean	0	0.600	27.1	91.3	≤ 45.96	0.002*
	2	0.597	74.4	44.6	≤ 3.57	0.002*
	3	0.597	62.4	55.9	≤ 4.71	0.002*
	4	0.588	56.4	61.5	≤ 4.03	0.005*
	5	0.574	98.5	17.9	≤ 19.83	0.019*
	6	0.556	97.0	18.5	≤ 18.1	0.075
Standard deviation	0	0.676	58.6	71.8	≤ 30.47	< 0.001*
	2	0.703	69.2	71.3	≤ 72.05	< 0.001*
	3	0.703	69.2	69.2	≤ 69.05	< 0.001*
	4	0.683	73.7	64.1	≤ 68.25	< 0.001*
	5	0.639	62.4	68.2	≤ 60.8	< 0.001*
	6	0.589	80.5	39.5	≤ 72.42	0.005*
Entropy	0	0.546	63.9	45.1	≤ 4.66	0.149
	2	0.565	86.5	32.3	≤ 5.51	0.039*
	3	0.563	89.5	29.2	≤ 5.55	0.047*
	4	0.559	85.7	33.3	≤ 5.51	0.062
	5	0.540	82.7	35.9	≤ 5.46	0.215
	6	0.523	82.7	33.8	≤ 5.47	0.473
MPP	0	0.617	27.1	93.3	≤ 51.29	< 0.001*
	2	0.726	72.2	70.3	≤ 55.29	< 0.001*
	3	0.743	74.4	70.8	≤ 55.14	< 0.001*
	4	0.726	72.9	65.6	≤ 54.75	< 0.001*
	5	0.698	82.7	55.9	≤ 56.87	< 0.001*
	6	0.667	80.5	49.2	≤ 55.09	< 0.001*
Skewness	0	0.507	9.0	74.9	≤ -0.09	0.832
	2	0.509	98.5	10.3	≤ 0.63	0.777
	3	0.531	66.2	44.1	≤ -0.04	0.340
	4	0.553	56.4	57.9	> 0.04	0.105
	5	0.553	66.9	50.3	> -0.14	0.109
	6	0.549	55.6	62.6	> -0.11	0.140
Kurtosis	0	0.546	76.7	38.5	≤ 1.83	0.155
	2	0.572	74.4	44.1	> 0.44	0.024*
	3	0.554	71.4	42.1	> -0.06	0.095
	4	0.515	9.0	99.0	> 6.38	0.647
	5	0.503	88.7	1.0	≤ 4.77	0.938
	6	0.505	87.2	3.6	≤ 3.31	0.871

**Figure 2:**
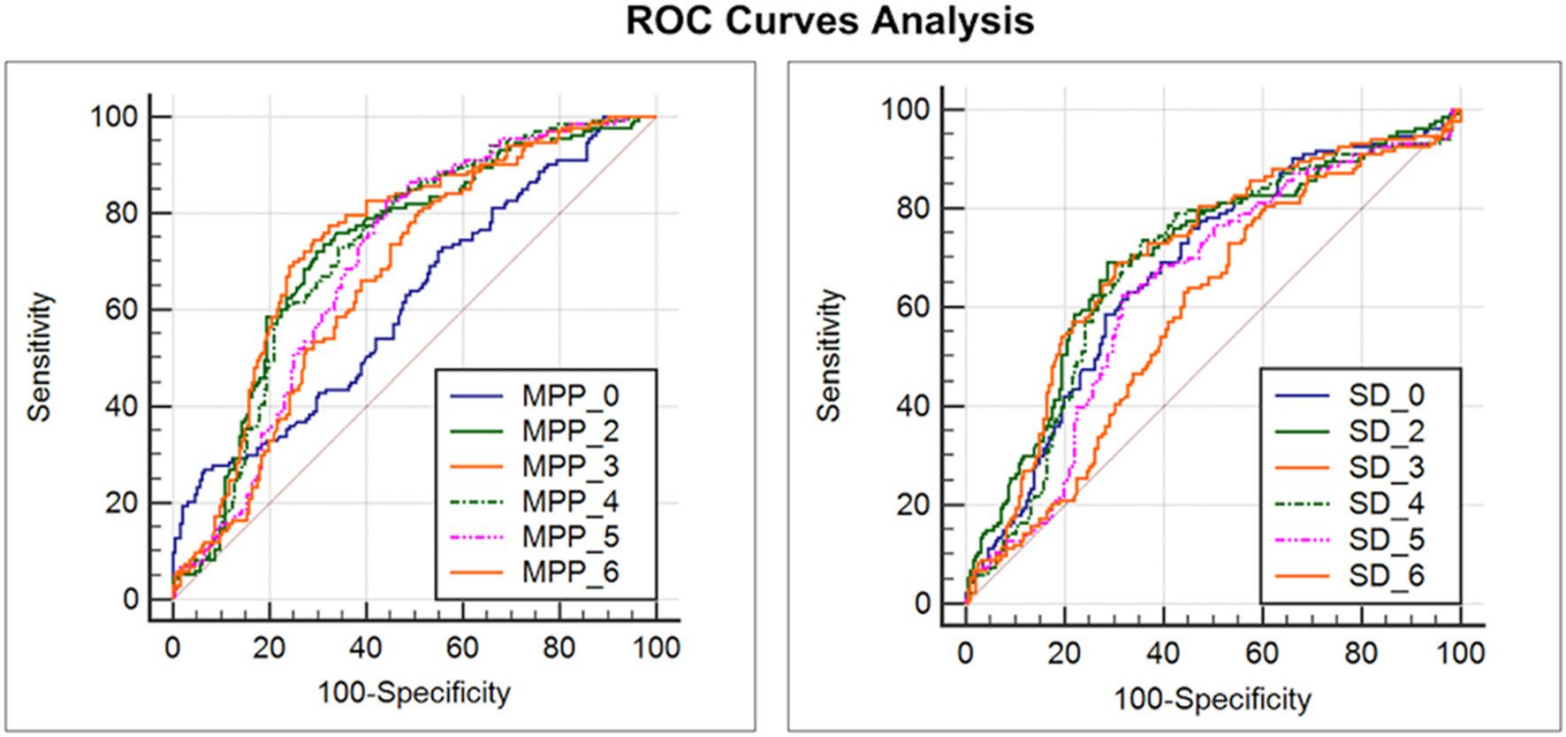
Figure shows the graphical representation of receiving operator curve (ROC) of pretreatment texture parameters with the best area under the curve (AUC), in particular is possible to appreciate standard deviation and MPP parameters as follows: MPP with Spatial Filter Scale (SSF) 2, 3 and 4 showed an AUC of 0.726, 0.743 and 0.726 respectively (all P < 0.001) and a sensitivity of 72.2, 74.4 and 72.9 and specificity of 70.3, 70.8 and 65,6 respectively. Otherwise, both Standard Deviation at SFF 2 and 3 found an AUC of 0.70 (all P < 0.001) with sensitivity of 69.2 for SSF 2 and 3 and specificity of 71.3 and 69.2 respectively.

Among them, *Standard Deviation* and *MPP* represent the two texture parameters, with good AUC; in particular *MPP* with SSF 2, 3 and 4 showed AUC of 0.726, 0.743 and 0.726 respectively (all *P* < 0.001), sensitivity of 72.2, 74.4 and 72.9 and specificity of 70.3, 70.8 and 65.6 respectively. Otherwise, both *Standard Deviation* at SFF 2 and 3 found an AUC of 0.70 (all *P* < 0.001) with sensitivity of 69.2 for SSF 2 and 3 and specificity of 71.3 and 69.2 respectively (Fig. 2).

**Figure 3:**
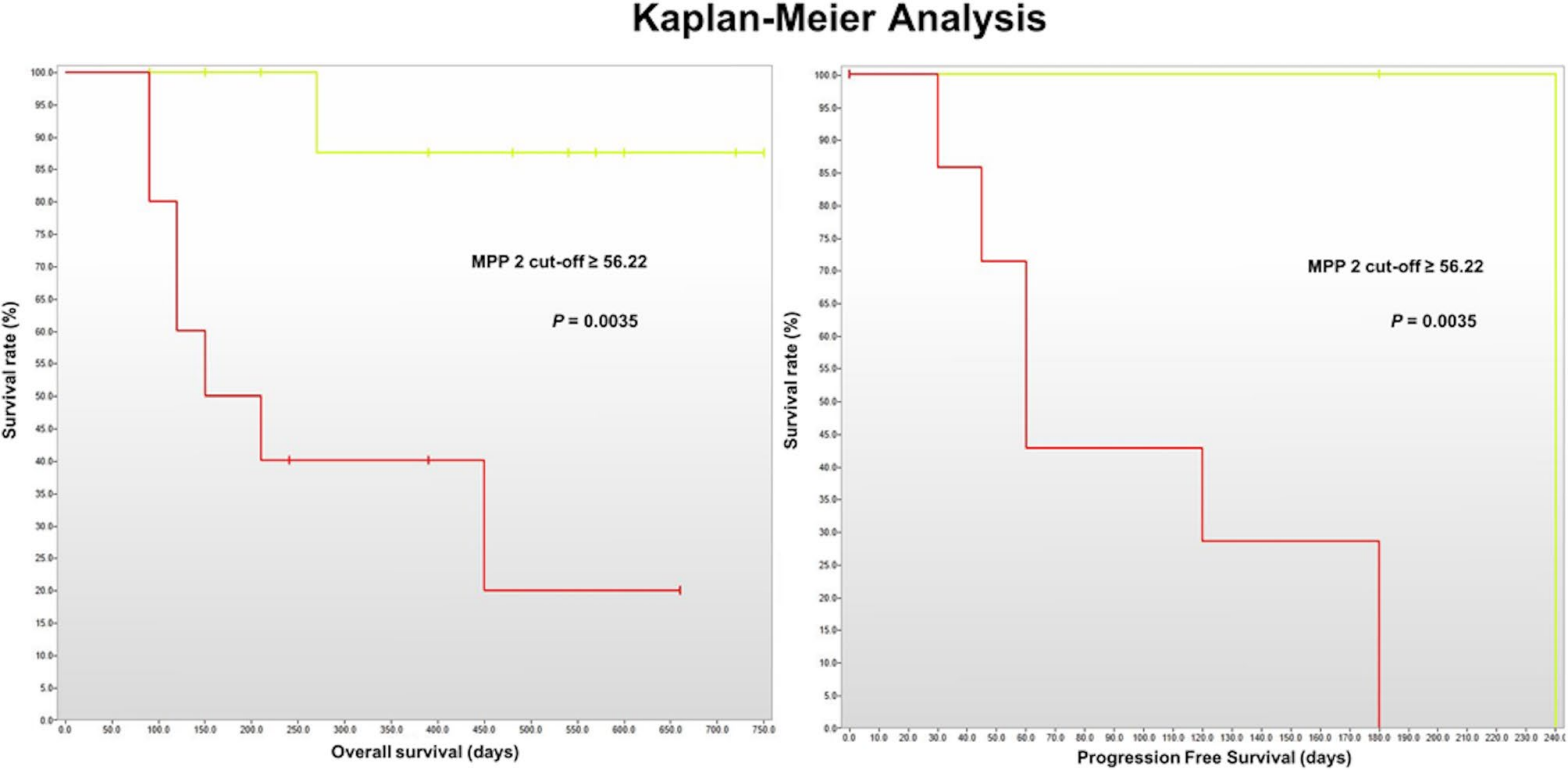
Figure shows results of Kaplan–Meier analysis for MPP Texture parameters at Spatial Filter Scale (SSF) 2 for Overall Survival (OS) and Progression Free Survival (PFS). When dichotomized at the optimal threshold identified in Kaplan–Meier analysis, MPP under 56.22 at fine scale (SSF = 2) was significantly associated with lower OS and PFS after administration of Pembrolizumab (red line), while MPP above 56.22 is significantly associated with higher OS and PFS (green line), *P* = 0.0035 both.

**Figure 4:**
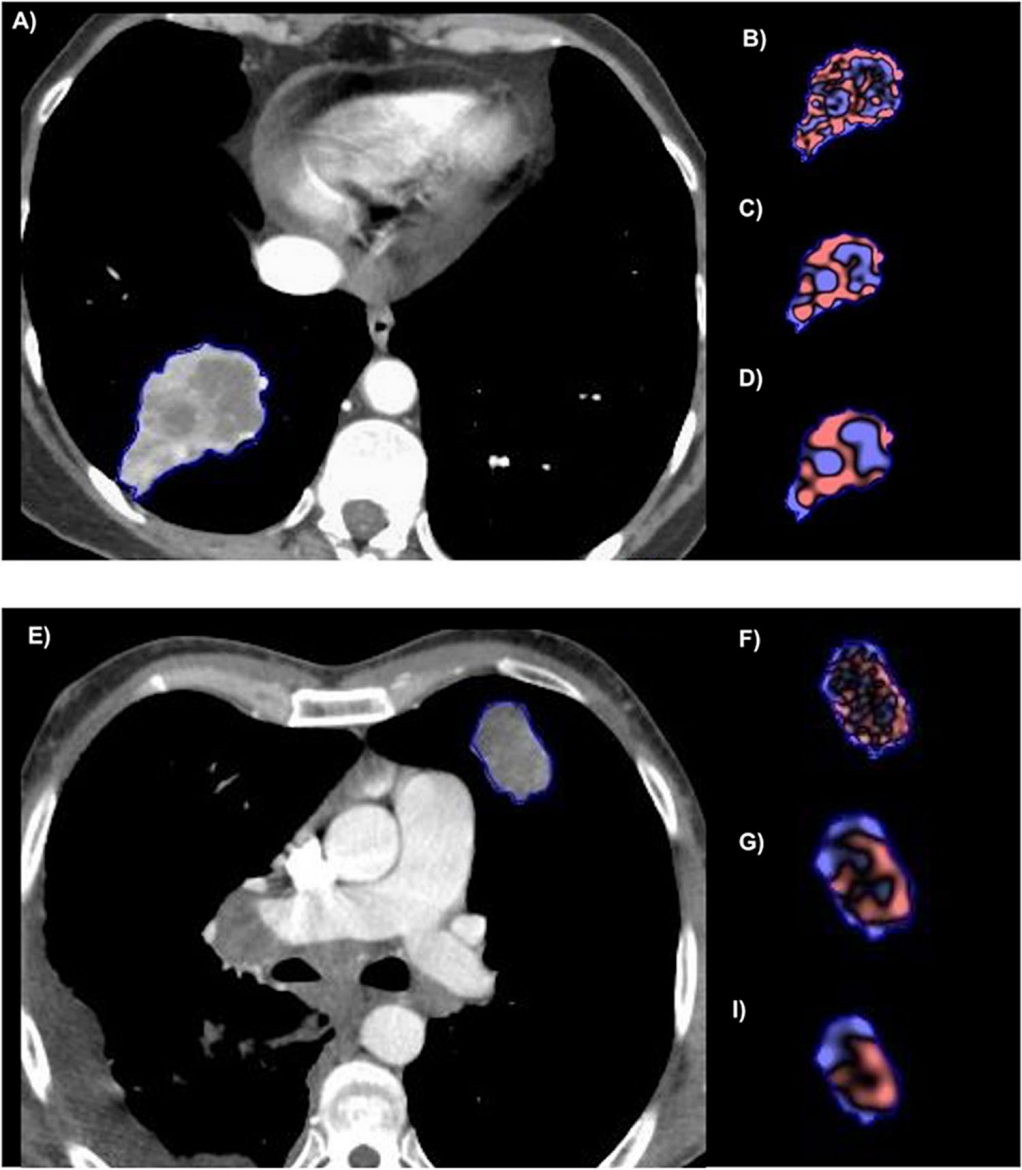
Picture shows two explanatory cases of pretreatment contrast enhanced CT at portal-venous phase and the associated Texture analysis extracted with a dedicated software (TexRAD version 3.2.0, Feedback Medical Ltd,Cambridge, UK; https://fbkmed.com/texrad-landing-2/). (**A**) shows a patient with good prognosis with an Overall Survival (OS) of 24 months and partial response to treatment while (**E**) represents a patient with poor prognosis, an OS of 3 months and a progression free survival (PFS) of two months concluded with death; Texture analysis at different spatial scale filters (SSF) is shown for fine SFF in (**B**,**F**), medium in (**C**,**G**) and coarse in (**D**,**I**). In line with the cut-off of 56.22 found on the Kaplan–Meier analysis, above which patients show good OS and PFS: patient reported in (**A**), with excellent OS had an MPP value at SSF 2 of 74.76 while patient depicted in (**E**) with poor OS had an MPP value at SFF 2 of 38,97.

Then, the texture parameters with significant AUC and the abovementioned values of sensitivity and specificity where combined with OS and PFS for Kaplan–Meier analysis. When dichotomized at the optimal threshold identified in Kaplan–Meier analysis, *MPP* under 56.22 at fine scale (SSF = 2; *P* = 0.0035) was significantly associated with lower overall survival and progression-free survival time after administration of Pembrolizumab (Figs. 3 and 4).

Cox regression analysis applied for the TA of pretreatment CECT demonstrated marked associations with OS and *MPP* at medium scale SFF 4 (*P* = 0.0038; HR = 0.89 [CI 95%: 0.83,0.96]).

## Discussion

Our results demonstrate that texture features of IV stage NSCLC on baseline CECT images are associated with prediction of OS and PFS in patients treated with an anti-PD-1 therapy, such as Pembrolizumab. In fact, pretreatment *MPP* values at fine and medium filter showed a good prognostic value, with an AUC of 72% and 74% respectively (all *P* values < 0.001). Interestingly, the further performed analysis confirmed the trend express on the ROC curves for *MPP* at fine filters with an important added value: the possibility to have a selected texture parameter with cut-off of 56.2 able to discriminate with significance (P < 0.0035) OS and PFS in patients before the beginning of immunotherapy. As well as for the exploratory Cox regression analysis, *MPP* at medium filter scale, showed very promising results as independent factor of OS in the selected population (*P* = 0.0038; HR = 0.89 [CI 95%: 0.83,0.96]).

Our results reflected the implication between Radiomics as Imaging biomarker and tumor heterogeneity. In fact, a correlations between micro-structural heterogeneity observed at pathology and ultra-structure analysis of medical Imaging pixels has been highlighted by several Authors in different oncologic fields^11,25,26^. In particular, our results pointed out how pretreatment ROC analysis showed good AUC in parameters at fine and medium filter scale such as *Standard Deviation* and *MPP* with values above 70% in the prediction of disease progression. Results are still in a mild range of performance compared to other biomarkers that reach higher percentage as blood circulating biomarker i.e. neutrophil to lymphocyte ratio^27^, but, a confirmed biomarker associated with survival is not already validated, and the strong significance of the *P* value is encouraging and it would be interesting to expand the study population to assess how these parameters behave with more data.

The recurrent associated parameter called *MPP* represents a secondary histogram parameter that has the peculiarity of reducing the impact of dark objects of the images on the mean histogram value^22^; in other words it allows the extrapolation of the mean values of brightness of positive pixels in a ROI or VOI highlighted by filtration scales. Ganeshan B. and colleagues, in a previous study on NSCLC^11^, showed how the texture feature *MPP* has strong correlation with a peculiar characteristic of the tumor detected by the staining with pimonidazole, an exogenous marker of hypoxia. Moreover, *MPP* also correlates inversely with tumor CD34 expression which is a marker of angiogenesis expressed both on hematopoietic and somatic cells^28^. Similar results were also confirmed by another interesting study performed by Hayano K. and colleagues where Authors assessed the performance of predicting survival with TA in patients with NSCLC treated with antiangiogenetic chemotherapy and assessed with both CT and PET/CT^29^. Results showed how *MPP* and *Entropy* are related to OS and PFS and in particular Kaplan-Meyer analysis output that patients with a *MPP* lower than 26.36 showed a worse PFS and OS with a medium texture scale (radius 4 mm). Our results are in line with the trend of the latter study reported. The possible explanation of a different cut-off value might lay in the fact that Hayano K. and colleagues performed TA on unenhanced CT images, whereas in our study we CECT images obtained during the venous phase. As our study shows, the correlation between *MPP* and angiogenesis could be an expression of the different bloody supply of the tumor associated with a different immunologic response.

Texture analysis as already described, is a novel technique sensitive to different acquisition parameters including kV, percentage of iterative reconstructions, slice thickness and not least the use of contrast medium and the post-contrast phase chosen to perform the TA studied^11,30,31^. In particular, the use or not of contrast medium enables to highlight some tissue characteristics such as the blood supply of a tumor. For these reasons it is possible to explain the different cut-off emerged between the cited studies. On the contrary, it seems very important to underline another remarkable issue in common among the studies cited: the filters scale ranging between radii 1.8 mm and 4 mm of *MPP* show the best performances, highlighting the importance of the filters to enhance the heterogeneity within a tumoral tissue. This aspect results important due to the actual lack of standardization in this novel field, and the recurrence of comparable results on the same tumor type, let us to delineate through the studies some characteristics of the Texture parameters applicable in the future in clinical practice. That it would be very helpful not only for the characterization of the tumor ultra-structure itself, but also in the understanding of how the tumor interacts with new therapies such as immunotherapy.

Of course, an excessive simplification of the tumor cells biology mechanisms and the tumoral environment interactions with therapies is not possible, but TA show in different studies conducted on different type of tumors such interesting results in terms of prediction of survival. An example is given by the correlation of TA with survival in colorectal liver metastases patients treated with Regorafenib^15^, where *Entropy*, *Uniformity* and *Standard Deviation* resulted associated with OS. Defour L. B. and colleagues showed also how TA such as *Kurtosis* and *Skewness* are predictor of OS in pre-surgical CECT in hepatocellular cancer patients^32^. As mentioned before, Durot C. and collegues find a cut-off for *Skewness* in pretreatment CECT to assess PFS and OS in patients with metastatic melanoma treated with Pembrolizumab^14^. Several studies have been performed regarding lung cancer and prediction of survival with TA and Radiomic since now^33–37^ but often the comparability among studies is affected by different imaging techniques, different Texture parameters assessed (first, second or higher orders) and different software that still need larger validation. However, the importance of this emerging Imaging biomarkers is related to the possible future clinical application, in the era of Precision Medicine. In fact, the added value of an Imaging biomarker in the clinical set, could help the decision-making process in order to select the best treatment for the specific patient, even if standardization process is still a limitation for a clinical setting^38,39^.

Despite the interesting results, our study has some limitations: (a) small sample size; (b) relatively short follow-up due to the recent approval of Pembrolizumab as first-line therapy in advanced NSCLC; (c) texture analysis was performed only with first order statistical method; (d) due to the inoperable stage of NSCLS included in the study, the possible bias that texture features extracted reflects tumor’s overall aggressiveness rather than biomarkers of response to immunotherapy related to the lack of global immune-phenotype analysis on baseline and follow-up; (e) absence of an inter-reader agreement analysis; (f) the lack of a multivariate analysis including different clinical and histopathological data to include in an Artificial intelligence approach also with a training and validation cohorts.

## Conclusion

In conclusion, the results of our preliminary experience show how first-order texture parameter, could potentially represent a survival imaging biomarker in patients with advanced NSCLC treated with first-line Pembrolizumab. Further study with larger cohorts are needed to confirm the results for clinical setting.

## Data Availability

The datasets during and/or analyzed during the current study available from the corresponding author on reasonable request.

## Abbreviations

ALK: Anaplastic lymphoma kinase
AUC: Area under the curve
CECT: Contrast enhanced computed tomography
EGFR: Epidermal growth factor receptor
ICIs: Immune-checkpoints inhibitors
NSCLC: Non-small cell lung cancer
OS: Overall survival
PD-1/PD-L1: Programmed cell death protein-1/programmed cell death-ligand 1
PFS: Progression-free survival
ROC: Receiver operating curve
ROI: Region of Interest
SSF: Spatial scale filter
TA: Texture analysis
TKIs: Tyrosine kinase inhibitors
VOI: Volume of interest
